# Concerns about unapproved meningococcal vaccination for eculizumab therapy in Japan

**DOI:** 10.1186/1750-1172-9-48

**Published:** 2014-04-10

**Authors:** Tetsuya Tanimoto, Eiji Kusumi, Kazutaka Hosoda, Kaduki Kouno, Tamae Hamaki, Masahiro Kami

**Affiliations:** 1 Department of Internal Medicine, Navitas Clinic, 3-1-1, Shibasaki-cho, 190-0023 Tachikawa, Tokyo, Japan; 2 Division of Social Communication System for Advanced Clinical Research, Institute of Medical Science, University of Tokyo, 4-6-1, Shirokanedai, Minato-ku 108-8639, Tokyo, Japan

**Keywords:** Paroxysmal nocturnal hemoglobinuria, Atypical hemolytic uremic syndrome, Eculizumab, Meningococcal vaccines, Regulatory authority, Japan

## Abstract

An orphan medicinal product, eculizumab is approved in Japan and globally for treating paroxysmal nocturnal hemoglobinuria and atypical hemolytic uremic syndrome. Eculizumab therapy can cause late complement pathway deficiencies that predispose patients to meningococcal infections. Although meningococcal vaccinations are typically considered mandatory for eculizumab therapy, no approved vaccine is available in Japan as of March, 2014. Advertising unapproved, privately imported pharmaceuticals is prohibited under Japanese pharmaceutical law; detailed information concerning the unapproved meningococcal vaccines is therefore not widely available. The situation jeopardizes the safety of patients receiving eculizumab therapy, and Japanese clinicians are advised caution when prescribing this therapy.

## Letter to the Editor

The Japanese vaccine policy has been criticized for its lack of a rational scientific approach [1]. The Japanese regulatory authority has its own policies for pharmaceutical regulatory reviews and approvals; the gap between Japan and the United States (US) and the European Union regarding approval of novel therapeutics has become a major issue in Japan [2]. For example, the Japanese government continued to support use of a domestically manufactured oral (live) polio vaccine until 2012, and only reluctantly permitted importation of an inactivated polio vaccine from a foreign manufacturer due to growing public criticism [3]. Although these issues have improved in recent years, a number of vaccines approved in other countries remain unavailable in Japan. Here we describe the problem of a lack of approved meningococcal vaccines, which has led to serious confusion among hematology and infectious disease specialists, and has contributed to serious safety concerns for patients in Japan.

An orphan medicinal product, eculizumab (Soliris, Alexion Pharmaceuticals) is a recombinant, fully humanized hybrid IgG2/IgG4 monoclonal antibody directed against human complement component C5; inhibition of complement activation at the level of C5 creates a functional C5 deficiency [4]. The drug first obtained regulatory approval in the US in March 2007 to reduce hemolysis associated with paroxysmal nocturnal hemoglobinuria (PNH); in September 2011, it was also approved for inhibition of complement-mediated thrombotic microangiopathy in patients with atypical hemolytic uremic syndrome (aHUS) [5]. Eculizumab is currently approved globally; Japanese regulatory approvals were obtained in April 2010 and September 2013 for treatment of PNH and aHUS, respectively [6,7].

It should be noted that late complement pathway deficiencies caused by eculizumab therapy predispose patients to meningococcal infections due to the absence of meningococcal lysis through classical and alternative pathways. In clinical trials and extension studies of eculizumab in PNH, two cases of meningococcal sepsis were reported during treatment, an infection rate of 0.42 per 100 patient-years [8]. Meningococcal infections can rapidly become life-threatening or fatal without appropriate management. Thus, it is essential to protect against meningococcal disease by administering vaccines against *Neisseria meningitides* to induce antibody-mediated killing of the microorganism. In prescribing information provided by the US Food and Drug Administration, the boxed warnings recommend that clinicians “comply with the most current Advisory Committee on Immunization Practices (ACIP) recommendations for meningococcal vaccination in patients with complement deficiencies,” and “immunize patients with a meningococcal vaccine at least 2 weeks prior to administering the first dose of Soliris, unless the risks of delaying Soliris therapy outweigh the risks of developing a meningococcal infection [5].” Similarly, in the summary of product characteristics from the European Medicines Agency, patients who are not currently vaccinated against *Neisseria meningitides* are contraindicated to receive eculizumab therapy; the Agency provides clear statements that “to reduce the risk of infection, all patients must be vaccinated at least 2 weeks prior to receiving Soliris. PNH patients must be vaccinated 2 weeks prior to Soliris initiation. aHUS patients who are treated with Soliris less than 2 weeks after receiving a meningococcal vaccine must receive treatment with appropriate prophylactic antibiotics until 2 weeks after vaccination. Patients must be re-vaccinated according to current medical guidelines for vaccination use. Tetravalent vaccines against serotypes A, C, Y and W135 are strongly recommended, preferably conjugated ones [9].”

However, no recommendation for meningococcal vaccination appears in the Japanese package insert, although a statement about the risk of meningococcal infection appears in the boxed warnings [10]. This is because there is no approved meningococcal vaccine in Japan as of March 2014, and advertisement of unapproved pharmaceuticals is prohibited under Japanese pharmaceutical law. However, even in Japan, patients in eculizumab clinical trials received meningococcal vaccination [6,7]. Therefore, it has become a common practice for Japanese hematology specialists to provide unapproved, privately imported meningococcal vaccines through travelers’ clinics for overseas tourists. This unofficial and unendorsed solution has caused confusion among physicians and patients because detailed treatment information is not shared through official routes. Although we had administered dozens of meningococcal vaccinations to patients at our travelers’ clinic, none of the physicians administering eculizumab therapy provided information on which meningococcal vaccine the patients should receive (polysaccharide or polysaccharide-protein conjugate), the number of doses, or at what intervals the patients should be re-vaccinated. Furthermore, we have concerns about the costs of unapproved vaccination (up to several hundred US dollars), which are not compensated by the universal insurance coverage system. Considering the enormous financial burden of eculizumab therapy, around 30,000 to 50,000 US dollars per month, it seems ironic that vaccination is not compensated. The current Japanese vaccine regulatory policy must be reformed as soon as possible, and Japanese clinicians should adopt ACIP recommendations without age limitations for Japanese patients with persistent complement component deficiencies that result from eculizumab treatment [11].

## Abbreviations

PNH: Paroxysmal nocturnal hemoglobinuria; aHUS: Atypical hemolytic uremic syndrome; ACIP: Advisory Committee on Immunization Practices.

## Competing interests

The authors declare that they have no competing interests.

## Authors’ contributions

TT, EK, and MK contributed to the conception of this letter. All authors have been involved in drafting the manuscript and critically reviewing it for important intellectual content and have given final approval of this version of the manuscript.

## References

[B1] Shibuya K Hashimoto H Ikegami N Nishi A Tanimoto T Miyata H Takemi K Reich MR Future of Japan’s system of good health at low cost with equity: beyond universal coverage Lancet 2011 378 1265 1273 10.1016/S0140-6736(11)61098-2 21885100

[B2] Hirai Y Kinoshita H Kusama M Yasuda K Sugiyama Y Ono S Delays in new drug applications in Japan and industrial R&D strategies Clin Pharmacol Ther 2010 87 212 218 10.1038/clpt.2009.215 19940847

[B3] Tanimoto T Murashige N Hosoda M Kusumi E Ono S Kami M Shibuya K Vaccination for whom? Time to reinvigorate Japanese vaccine policy Lancet 2012 380 1647 23141615 10.1016/S0140-6736(12)61948-5

[B4] Luzzato L Gianfaldoni G Notaro R Management of paroxysmal nocturnal haemoglobinemia: a personal view Br J Haematol 2011 153 709 720 10.1111/j.1365-2141.2011.08690.x 21517820

[B5] U.S. Food and Drug Administration Prescribing information for Soliris http://www.accessdata.fda.gov/drugsatfda_docs/label/2011/125166s172lbl.pdf

[B6] Pharmaceuticals and Medical Devices Agency Review report of Soliris for paroxysmal nocturnal hemogulobinuria. (in Japanese) http://www.info.pmda.go.jp/shinyaku/P201000032/870056000_22200AMX00316000_A100_1.pdf

[B7] Pharmaceuticals and Medical Devices Agency Review report of Soliris for atypical hemolytic uremic syndrome. (in Japanese) http://www.info.pmda.go.jp/shinyaku/P201300125/870056000_22200AMX00316000_A100_1.pdf

[B8] Hillmen P Muus P Röth A Elebute MO Risitano AM Schrezenmeier H Szer J Browne P Maciejewski JP Schubert J Urbano-Ispizua A de Castro C Socié G Brodsky RA Long-term safety and efficacy of sustained exulizumab treatment in patients with paroxysmal nocturnal haemoglobinuria Br J Haematol 2013 162 62 73 10.1111/bjh.12347 23617322 PMC3744747

[B9] European Medicines Agency Soliris: EPAR – Product Information http://www.ema.europa.eu/docs/en_GB/document_library/EPAR_-_Product_Information/human/000791/WC500054208.pdf

[B10] Pharmaceuticals and Medical Devices Agency The package insert of Soliris. (in Japanese) http://www.info.pmda.go.jp/downfiles/ph/PDF/870056_6399424A1023_1_03.pdf

[B11] Centers for Disease Control and Prevention Prevention and Control of Meningococcal Disease: Recommendations of the Advisory Committee on Immunization Practices (ACIP) http://www.cdc.gov/mmwr/preview/mmwrhtml/rr6202a1.htm

